# Ipriflavone-Loaded Mesoporous Nanospheres with Potential Applications for Periodontal Treatment

**DOI:** 10.3390/nano10122573

**Published:** 2020-12-21

**Authors:** Laura Casarrubios, Natividad Gómez-Cerezo, María José Feito, María Vallet-Regí, Daniel Arcos, María Teresa Portolés

**Affiliations:** 1 Departamento de Bioquímica y Biología Molecular, Facultad de Ciencias Químicas, Universidad Complutense de Madrid, Instituto de Investigación Sanitaria del Hospital Clínico San Carlos (IdISSC), 28040 Madrid, Spain; laura.casarrubios.molina@gmail.com (L.C.); mjfeito@ucm.es (M.J.F.); 2 Departamento de Química en Ciencias Farmacéuticas, Facultad de Farmacia, Universidad Complutense de Madrid, Instituto de Investigación Sanitaria Hospital 12 de Octubre i+12, Plaza Ramón y Cajal s/n, 28040 Madrid, Spain; magome21@ucm.es; 3 CIBER de Bioingeniería, Biomateriales y Nanomedicina, CIBER-BBN, 28040 Madrid, Spain

**Keywords:** endocytosis, ipriflavone, mesoporous nanospheres, nanoparticles, oxidative stress, pre-osteoblasts

## Abstract

The incorporation and effects of hollow mesoporous nanospheres in the system SiO_2_–CaO (nanoMBGs) containing ipriflavone (IP), a synthetic isoflavone that prevents osteoporosis, were evaluated. Due to their superior porosity and capability to host drugs, these nanoparticles are designed as a potential alternative to conventional bioactive glasses for the treatment of periodontal defects. To identify the endocytic mechanisms by which these nanospheres are incorporated within the MC3T3-E1 cells, five inhibitors (cytochalasin B, cytochalasin D, chlorpromazine, genistein and wortmannin) were used before the addition of these nanoparticles labeled with fluorescein isothiocyanate (FITC–nanoMBGs). The results indicate that nanoMBGs enter the pre-osteoblasts mainly through clathrin-dependent mechanisms and in a lower proportion by macropinocytosis. The present study evidences the active incorporation of nanoMBG–IPs by MC3T3-E1 osteoprogenitor cells that stimulate their differentiation into mature osteoblast phenotype with increased alkaline phosphatase activity. The final aim of this study is to demonstrate the biocompatibility and osteogenic behavior of IP-loaded bioactive nanoparticles to be used for periodontal augmentation purposes and to shed light on internalization mechanisms that determine the incorporation of these nanoparticles into the cells.

## 1. Introduction

Bioactive glasses are a group of bioceramics that exhibit bone regeneration properties. Since their discovery in 1971, over 1.5 million patients have been treated with Bioglass 45S5, the original four-component Bioglass composition (45 wt % SiO_2_, 24.5 wt % CaO, 24.5 wt % Na_2_O, 6 wt % P_2_O_5_). In addition to orthopedic surgery as bone graft substitutes, bioactive glasses applications in dentistry involve their use as dental restorative materials, mineralizing agents, coating material for dental implants, pulp capping and root canal treatment [1]. The first particulate form of Bioglass, trademark PerioGlass^®^, in 1993, is still sold for the treatment of periodontal defects and has become a standard for the treatment of these types of clinical defects [2].

The research developed during the subsequent decades has resulted in new materials that significantly differs from the original melt-derived Bioglass 45S5. The use of the sol–gel process in the 1990s [3,4], the preparation of bioactive star gels [5] and the development of mesoporous bioactive glasses (MBG) revealed new potential applications in the field of bone tissue regeneration and drug delivery platforms [6,7,8]. Compared to conventional bioactive glasses, MBGs exhibit higher surface area and porosity, which give them excellent drug-loading ability and superior bone-forming capacity [9,10,11]. These characteristics make MBGs very attractive as bone-graft material to be used in the regeneration of periodontal bone defects since they can augment the height and bone volume of the alveolar ridge for the insertion of dental implants, whereas they can deliver antibiotic or antiosteoporotic drugs to prevent infection or promote bone healing in the case of patients with diminished bone-forming capability, respectively.

The advances in nanomedicine have opened new research lines involving the synthesis and development of nanoparticles, including carbon-based nanomaterials, hydroxyapatite, iron oxide, zirconia, silica, silver or titania, among others [12]. Thus, nanodentistry is a consequence of the progress in nanomaterials, tissue engineering and nanomedicine, being very beneficial for diagnostic procedures, treatment and prevention of oral and dental diseases. Currently, the use of nanoparticles in dentistry comprises dental filling, reinforcement of dental implants, polishing of enamel surface, prevention of caries, teeth whitening and anti-sensitivity agents [13]. In this sense, recent advances have been made with gold nanoparticles as a biomaterial in dentistry due to their antifungal and antibacterial activity, mechanical properties and availability of different sizes and concentrations [14]. However, the studies focused on the use of bioactive nanoparticles for periodontal bone augmentation are very scarce, and most of them have been carried out with hydroxyapatite nanoparticles [15,16]. In this context, the recent developments in the preparation of mesoporous bioactive glass nanoparticles could provide a very interesting alternative for this purpose [17,18,19,20]. On the other hand, the coupling of osteogenesis and angiogenesis is crucial in periodontal tissue regeneration and biomaterials loaded with different agents that act synergistically on both processes are very recently being designed to achieve periodontal regeneration [21].

One of the most interesting strategies to promote bone regeneration under osteoporotic conditions consists of loading bioactive materials with different drugs to treat osteoporotic bone by either promoting the osteogenesis process or inhibiting the activity of osteoclasts, or both [22,23]. Among the drugs used for this purpose, it has been shown that ipriflavone (IP) prevents osteoporosis by inhibiting bone resorption [24]. On the other side, oral administration of IP (1200 mg daily) to subjects diagnosed of primary hyperparathyroidism indicated that this drug has great potential in the therapy of metabolic bone pathologies in which there is high bone turnover [25]. As a nanotherapeutic strategy, different inorganic nanoparticles have been designed for drug incorporation and intraosseous administration in osteoporosis and regenerative therapies for bone diseases [26,27]. This type of administration, with nanoparticles loaded with drugs that will be released inside the bone cells, allows significantly reducing the quantity of drug required to carry out the desired effect.

In the present work, we have evaluated the effects of mesoporous bioactive nanospheres (nanoMBGs) loaded with IP on MC3T3-E1 osteoprogenitor cells, the most relevant model of *in vitro* osteogenesis [28], as a nanotherapeutic strategy to promote bone regeneration. The rationale behind this selection is the osteogenic potential and drug delivery capabilities of nanoMBGs, which could provide an excellent strategy as a bone graft for periodontal defects and also for the treatment of infections and inflammatory processes such as those that occur in periodontitis. These nanospheres are synthesized in the ternary system SiO_2_–CaO–P_2_O_5_ and have shown excellent *in vitro* bioactive behavior in previous studies [29]. Since the effectiveness of treatment with nanoparticles designed for intracellular drug release depends on their efficient incorporation into cells, we have investigated the mechanisms of incorporation of these nanospheres into pre-osteoblasts. Thus, to identify the endocytic mechanisms by which these nanoMBGs are incorporated within the MC3T3-E1 cells, five inhibitors (cytochalasin B, cytochalasin D, chlorpromazine, genistein and wortmannin) were used before the addition of these nanoparticles labeled with fluorescein isothiocyanate (FITC–nanoMBGs). On the other hand, to assess the intracellular action of the drug, the effects of unloaded and IP-loaded nanospheres (nanoMBG–IPs) on MC3T3-E1 pre-osteoblasts were evaluated in a comparative study by analyzing the following cellular parameters: cell viability, apoptosis, cell cycle, intracellular content of reactive oxygen species, intracellular content of Ca^2+^, production of interleukin 6, alkaline phosphatase activity and matrix mineralization. The study of all these parameters is focused on testing the absence of cytotoxicity of nanoMBG–IPs and their potential as a nanotherapeutic strategy for the intracellular delivery of ipriflavone to promote osteogenesis in the periodontal defects. The final aim of this study is to demonstrate the biocompatibility and osteogenic behavior of nanoMBG–IP and to shed light on the mechanisms that rule the incorporation of these nanoparticles into the cells.

## 2. Materials and Methods

### 2.1. Preparation, Characterization and Labeling of Mesoporous SiO_2_–CaO Nanospheres

Mesoporous SiO_2_–CaO–P_2_O_5_ nanospheres (nanoMBGs) were synthesized following the method described in previous work [30]. This method consists of the preparation of an O/W emulsion where two different templates are dissolved. Briefly, poly(styrene)-block-poly(acrylic acid) (PS-b-PAA) with average Mw = 38,000, was dissolved in tetrahydrofuran (THF) and poured on a hexadecyltrimethylammonium bromide (CTAB) water solution. Then, the appropriated amounts of Ca(NO_3_)_2_·4H_2_O, triethyl phosphate (TEP) and tetraethyl orthosilicate (TEOS) were added dropwise dissolved in water and ethanol, respectively. After 24 h stirring, the product was collected by centrifugation, dried and calcined at 550 °C to remove the organic templates (see Supplementary Materials for a detailed description of the synthesis).

Scanning electron microscopy (SEM) and transmission electron microscopy (TEM) images were collected with a JEOL F-6335 microscope and a JEOL-1400 microscope (JEOL, Tokyo, Japan), respectively.

Textural properties were studied by means of nitrogen adsorption analysis using an ASAP 2020 equipment (Micromeritics, Norcross, GA, USA). For this aim, nanoMBGs were degassed at 150 °C for 15 h. Fourier-transform infrared spectroscopy (FT-IR) was carried out using a Nicolet Magma IR 550 spectrometer (Nicolet Instruments, Madison, WI, USA). In order to collect more information from the surface of the nanoparticles, the spectra were collected by means of the attenuated total reflectance (ATR) sampling technique. Thermogravimetric analysis (TGA) was performed using a TG/DTA Seiko SSC/5200 thermobalance (SEIKO instruments, Chiba, Japan). The samples were heated from 50 to 600 °C at a heating rate of 1 °C min^−1^, using α-Al_2_O_3_ as reference.

For fluorescein isothiocyanate (FITC)-labeling, aminopropyl triethoxysilane (APTES) was dissolved in ethanol. Subsequently, 0.6 mg of fluorescein isothiocyanate was added and stirred for 5 h. This solution was added dropwise on the nanoMBG particle suspension, and the labeled particles were washed and collected by centrifugation (see Supplementary Materials for a detailed description of the labeling).

### 2.2. Antiosteoporotic Drug Loading

Ipriflavone (IP) was chosen as an antiosteoporotic drug for this study. For this aim, 300 mg of IP (7-isopropoxy-3-phenyl-4H-1-benzopyran-4-one) were dissolved in 6 mL of acetone as previously reported [31]. Subsequently, 80 mg of nanoMBGs were poured on this solution and stirred in a rotatory incubator at 100 rpm for 24 h. Ipriflafone-loaded nanoparticles (nanoMBG–IP) were filtered and washed with acetone and water, thus removing the excess of IP physically adsorbed on the external surface.

### 2.3. Cell Culture of MC3T3-E1 Pre-Osteoblasts for FITC–NanoMBG Incorporation. Evaluation of the Endocytic Mechanisms for FITC-NanoMBG Cell Entry

Since MC3T3-E1 osteoprogenitor cells are the most relevant model of in vitro osteogenesis [28], this cell line was chosen to investigate the entry mechanisms of these mesoporous bioactive nanospheres labeled with FITC in undifferentiated osteoblasts. This cell line was kindly provided by Dr. B.T. Pérez-Maceda (CIB, CSIC, Madrid, Spain). On the other hand, in this study, we have analyzed the effects of these nanospheres loaded with ipriflavone on the differentiation of pre-osteoblasts into mature osteoblasts, as explained below. For FITC–nanoMBG incorporation studies, MC3T3-E1 pre-osteoblasts (10^5^ cells/mL) were seeded in 24 well culture plates with Dulbecco’s Modified Eagle’s Medium (DMEM, Sigma Chemical Company, St. Louis, MO, USA) with fetal bovine serum (FBS, Gibco, BRL, 10% *vol*/*vol*), 1 mM L-glutamine (BioWhittaker Europe, Verviers, Belgium) and antibiotics (200 μg penicillin and 200 μg streptomycin per mL, BioWhittaker Europe, Verviers, Belgium). Cells were cultured for 24 h in a 5% CO_2_ incubator at 37 °C, and different doses of FITC–nanoMBGs (10, 30 and 50 μg/mL) were added afterward into the culture medium and maintained several times. Cells were harvested with trypsin-EDTA (0.25%), and FITC–nanoMBG incorporation was quantified through flow cytometry. The FITC–nanoMBG fluorescence was detected in a FACScalibur Becton Dickinson flow cytometer with a 530/30 filter, exciting the sample at 488 nm. The data acquisition and flow cytometric analysis conditions were set through negative and positive controls using the CellQuest Program of Becton Dickinson and maintained for all measurements. A total of 10^4^ cells were analyzed in each sample in order to ensure a correct statistical significance.

To identify the endocytic mechanisms by which these FITC–nanoMBG nanospheres are incorporated within the MC3T3-E1 cells, the inclusion in the culture medium of several specific endocytosis inhibitors was carried out before adding the nanoparticles, maintaining the cells 2 h under these conditions. The endocytosis inhibitors were: 20 μM cytochalasin B (MP Biomedicals, Eschwege, Germany), 4 μM cytochalasin D (MP Biomedicals, Eschwege, Germany), 30 μM chlorpromazine (Enzo Life Sciences, Barcelona, Spain), 3.7 μM genistein (Enzo Life Sciences, Barcelona, Spain), and 23 μM wortmannin (Enzo Life Sciences, Barcelona, Spain). Then, the culture medium was changed by a fresh medium containing 50 µg/mL FITC–nanoMBGs and cells were maintained for 2 h at 37 °C in a 5% CO_2_ incubator. Finally, cells were collected with trypsin-EDTA (0.25%) and the FITC–nanoMBG incorporation in each case was quantified by flow cytometry as stated above. All the analyses were compared with their respective controls without inhibitors.

### 2.4. Cell Size and Complexity Analysis

To study the cell size and complexity, forward angle (FSC) and side angle (SSC) scatters were detected, respectively, in a FACScalibur Becton Dickinson flow cytometer. A total of 10^4^ cells were analyzed in each sample in order to ensure a correct statistical significance.

### 2.5. Cell Viability Studies

Cell viability was measured by adding 0.005% (wt/vol) propidium iodide (PI) in PBS (Sigma-Aldrich, St. Louis, MO, USA) into the samples to stain the dead cells. The PI exclusion indicates the plasma membrane integrity. PI fluorescence was detected in a FACScalibur Becton Dickinson flow cytometer (Becton Dickinson, San Jose, CA, USA) with a 530/30 filter, exciting the sample at 488 nm. A total of 10^4^ cells were analyzed in each sample in order to ensure a correct statistical significance.

### 2.6. Cell-Cycle Analysis and Apoptosis Detection by Flow Cytometry

Cells in 0.5 mL of PBS were mixed with 4.5 mL of ethanol 70% and maintained overnight at 4 °C. Cell suspensions were then centrifuged for 10 min at 310× *g* and resuspended in 0.5 mL of RNAsa solution containing 0.1% Triton X-100, 20 µg/mL of IP and 0.2 mg/mL of RNAsa (Sigma-Aldrich, St. Louis, MO, USA). After 30 min of incubation at 37 °C, PI fluorescence was detected in a FACScan Becton Dickinson flow cytometer with a 585/42 filter, exciting the sample at 488 nm. The CellQuest Program of Becton Dickinson was used to calculate the percentage of cells in each cycle phase: G_0_/G_1_ (growth), S (DNA synthesis) and G_2_/M (growth and mitosis). To quantify the cell apoptosis, the SubG_1_ fraction (cells with fragmented DNA) was evaluated. A total of 10^4^ cells were analyzed in each sample in order to ensure a correct statistical significance.

### 2.7. Intracellular Reactive Oxygen Species (ROS) Content

Cell suspensions were incubated for 30 min at 37 °C with 100 μM of 2′,7′-dichlorofluorescein diacetate (DCFH/DA, Serva, Heidelberg, Germany). DCFH/DA can penetrate the cells and can be hydrolyzed by cytosolic esterases, producing DCFH, which is instantly oxidized by ROS to DCF, highly fluorescent and whose fluorescence intensity depends directly on the intracellular content of reactive oxygen species (ROS). DCF fluorescence was measured in a FACScalibur Becton Dickinson flow cytometer with a 530/30 filter, exciting the sample at 488 nm. A total of 10^4^ cells were analyzed in each sample in order to ensure a correct statistical significance.

### 2.8. Confocal Microscopy Studies

Cells were cultured on circular glass coverslips with 50 μg/mL FITC–nanoMBGs in the culture medium for 24 h. Afterward, cells were fixed with p-formaldehyde (3.7%) and permeated, adding 500 μL of Triton-X100 (0.1% in PBS). After 20 min of incubation with BSA (1% in PBS), samples were stained with 100 μL of rhodamine-phalloidin 1:40, washed with PBS and stained with 100 μL of 4′,6-diamidino-2-phenylindole (3 × 10^−6^ M in PBS, DAPI, Molecular Probes, Inc., Eugene, OR, USA). Finally, samples were observed through a Leica SP2 confocal laser scanning microscope. The fluorescence of rhodamine and DAPI were excited at 540 and 405 nm, respectively, and detected at 565 and 420/586 nm, respectively.

### 2.9. Intracellular Calcium Content

After incubation of cell suspensions for 30 min with the probe Fluo4-AM (5 μM, Thermo Fisher Scientific, Madrid, Spain), which can penetrate the cells and be hydrolyzed by cytosolic esterases, Fluo4 fluorescence was measured in a FACScan Becton Dickinson flow cytometer with a 530/30 filter, exciting the sample at 488 nm. Finally, to check the assay sensitivity, A-23,187 ionophore (5 μM, Sigma-Aldrich, St. Louis, MO, USA) was added to each sample. A total of 10^4^ cells were analyzed in each sample in order to ensure a correct statistical significance.

### 2.10. Alkaline Phosphatase Activity

A total of 2 × 10^4^ cells/mL were cultured in 24 well plates and maintained for 24 h in a 5% CO_2_ incubator at 37 °C, with 1 mL/well of culture medium (DMEM with 10% FBS, 1 mM L-glutamine 1 mM and antibiotics), supplemented with 10 mM L-ascorbic acid and 50 μg/mL β-glycerolphosphate in order to promote cell differentiation. To evaluate the nanomaterial effects on alkaline phosphatase (ALP) activity, as a key indicator of osteoblast phenotype expression, 50 μg/mL of nanoMBGs with or without ipriflavone were added into the wells and cells were maintained for 11 days in a 5% CO_2_ incubator at 37 °C, refreshing the culture medium every 4 days. ALP activity was detected using Reddi and Huggins´ method (Reddi and Huggins, 1972, SpinReact S.A., Girona, Spain), and the obtained values were normalized with respect to total cell protein content, measured using Bradford’s method with bovine serum album (BSA) as standard.

### 2.11. Mineralization Assay

A total of 2 × 10^4^ cells/mL were seeded in 12 well plates and maintained for 24 h in a 5% CO_2_ incubator at 37 °C, with 1.5 mL/well of culture medium (DMEM with 10% FBS, 1 mM L-glutamine 1 mM and antibiotics), supplemented with 10 mM L-ascorbic acid and 50 μg/mL β-glycerophosphate in order to promote cell differentiation. Then, 50 μg/mL of nanoMBGs with or without ipriflavone were added into the wells and cells were maintained for 11 days in a 5% CO_2_ incubator at 37 °C, refreshing the culture medium every 4 days. Afterward, the culture medium was removed, and the cell cultures were treated with glutaraldehyde (10%) as a fixer for 1 h. Then, cells were stained with 40 mM Alizarin Red at pH 4.2 for 45 min in order to analyze the matrix mineralization. Finally, the stained extracellular deposits were dissolved with cetylpyridinium chloride (10% at pH 7), and the absorbance of the supernatants was measured at 620 nm.

### 2.12. Interleukin 6 (IL-6) Detection

The concentration of IL-6 secreted to the culture medium by 2 × 10^4^ cells/mL, after treatment with 50 μg/mL of nanoMBGs with or without ipriflavone, was measured using an ELISA IL-6 kit (Gen-Probe, Diaclone). This method is based on a sandwich ELISA where plates are pre-coated with a capture antibody highly specific for IL-6 and, after the incubation with the samples, a biotinylated secondary antibody is added, and the correct unions are revealed with streptavidin-avidin conjugated with horseradish peroxidase in a colorimetric reaction which is quantified in an ELISA Plate Reader at 450 nm, with a sensitivity of 10 pg/mL and an inter-assay variation coefficient <10%. Recombinant cytokine was adopted as standard.

### 2.13. Statistics

The results obtained appear as means of three replicate experiments plus their standard deviations, analyzed with the 22nd version of Statistical Package for the Social Sciences (SPSS). Statistical comparisons were carried out with the analysis of variance (ANOVA), and Scheffé and Games–Howell test was employed for post hoc analysis of differences between study groups, considering *p* < 0.005 as statistically significant.

## 3. Results and Discussion

### 3.1. Characterization of Mesoporous Nanospheres

**Figure 1:**
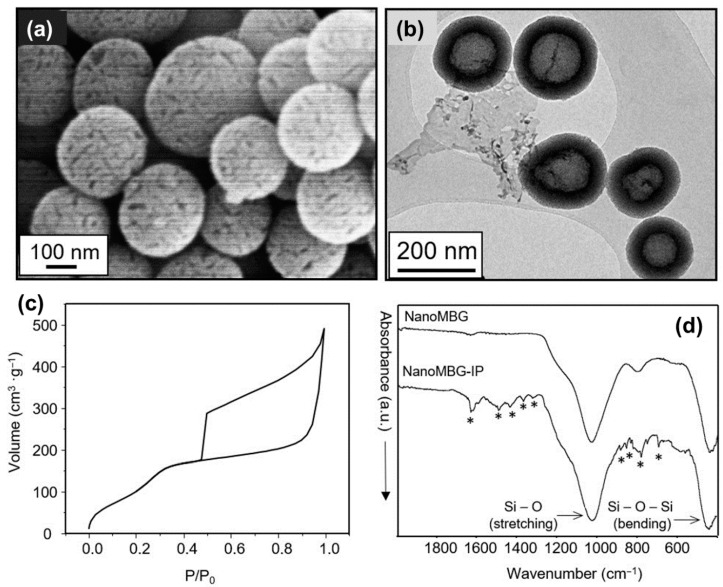
Characterization of mesoporous nanospheres. (**a**) scanning electron micrograph of hollow mesoporous nanospheres in the system SiO_2_–CaO (nanoMBG) spheres. (**b**) Transmission electron image of nanoMBG spheres. (**c**) Nitrogen adsorption/desorption isotherm of nanoMBG spheres. (**d**) FTIR spectra of nanoMBG and IP-loaded nanospheres (nanoMBG–IP) spheres (* indicates the absorption bands corresponding to ipriflavone).

Prior to any biological assay, the main physic-chemical features of the nanoparticles must be determined. For this purpose, electron microscopy (SEM and TEM) experiments, textural properties determination and FTIR analysis before and after drug-loading was carried out. Figure 1a shows an SEM image of nanoMBGs, pointing out that this material is made of non-aggregated spheres ranging in size between 150 and 250 nanometers. The spheres show porosity accessible to the external surface. TEM image (Figure 1b) provides more detailed information about the porous structure of nanoMBG spheres. The TEM image evidence that our spheres are composed of an inner cavity of about 100 nm in diameter, surrounded by a shell that exhibits a radial porosity. These two types of porosity are clearly reflected in the nitrogen adsorption/desorption isotherm shown in Figure 1c. The adsorption isotherm corresponds to a highly porous material with high surface area (see Table 1) and with a wide hysteresis loop type H2, characteristic of ink bottle-like pore as a clear reflection of the wide central cavity connected to the narrow necks of the radial pores of the shell. Finally, FTIR spectra evidence the presence of ipriflavone after the loading process (Figure 1d) with the characteristic absorption band of this compound (see Figure S1 in Supplementary Materials). Thermogravimetric analysis indicated 18% in weight of ipriflavone-load (see Figure S2 in Supplementary Materials), and the decrease of the textural parameters also evidence that the drug is filling or even occluding the pores of the spheres (Table 1).

### 3.2. Effects of NanoMBGs and NanoMBG-IPs on Size, Complexity, Apoptosis and Cell Cycle of MC3T3-E1 Pre-Osteoblasts

Once the main physic-chemical characteristics of nanoMBGs were determined, we proceeded to assess the potentially deleterious effects that these nanoparticles could exert on pre-osteoblast in terms of cell size, complexity, apoptosis or harmful variations in the cell cycle. No changes in pre-osteoblast size and complexity (FSC and SSC, respectively) were observed after the intracellular incorporation of nanoMBGs or nanoMBG–IPs (see Figure S3 in Supplementary Materials). In this context, we have observed in previous studies with MC3T3-E1 pre-osteoblasts that the incorporation of another type of nanoparticles, such as graphene oxide nanosheets, produced alterations as the increase in cell size (FSC) without changes in cell complexity (SSC) [32]. However, previous studies with RAW 264.7 macrophages and nanoMBGs evidenced a significant increase of macrophage complexity (SSC) after the treatment with these nanospheres due to their uptake by macrophages [29]. It is well known that these cell parameters, FSC and SSC, depend on different factors such as the cell surface and some organelles (lysosomes, mitochondria, nucleus or pinocytic vesicles) as well as on the presence of granulated material within the cell [33].

**Figure 2:**
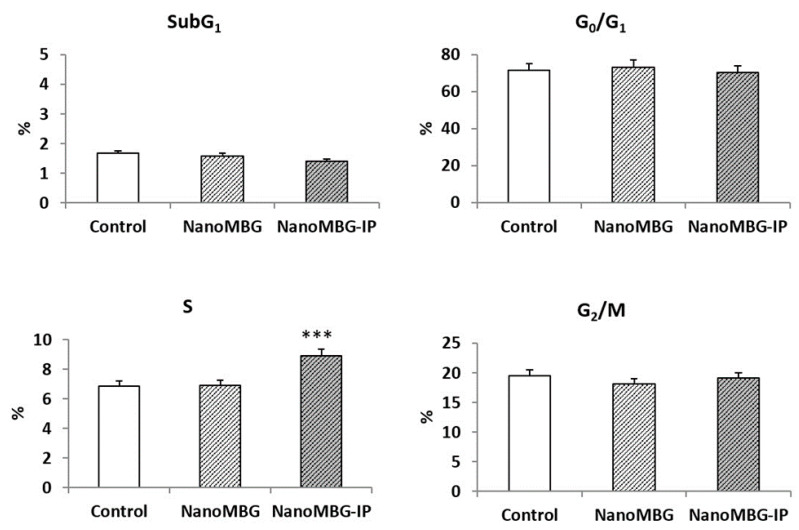
Effects of nanoMBGs and nanoMBG–IPs on cell cycle phases of MC3T3-E1 pre-osteoblasts and apoptosis percentage (Sub G_1_ fraction) after 24 h of treatment with 50 μg/mL of nanospheres. Control conditions without nanospheres were performed at the same time. Statistical significance: *** *p* < 0.005.

The effects of nanoMBG and nanoMBG–IP nanospheres on cell cycle phases (G_0_/G_1_, S and G_2_/M) of MC3T3-E1 pre-osteoblast and the percentage of cells in apoptosis (SubG_1_ fraction) were analyzed. Figure 2 shows that the treatment with 50 μg/mL of nanospheres without ipriflavone for 24 h did not induce alterations on G_0_/G_1_, S and G_2_/M phases. In the same way, nanoMBG–IPs did not induce changes in G_0_/G_1_ and G_2_/M phases. Nevertheless, a significant increment (*p* < 0.005) of the synthesis phase (S) was observed after the incubation of the MC3T3-E1 pre-osteoblasts with 50 μg/mL of nanoMBG–IPs, thus evidencing the protective impact of the ipriflavone into these cells. Moreover, nanoMBG and nanoMBG–IPs did not induce apoptosis on MC3T3-E1 pre-osteoblasts, detected as SubG1 fraction, in comparison with control cultures.

### 3.3. Effects of NanoMBGs and NanoMBG-IPs on Viability, Intracellular Reactive Oxygen Species (ROS) and Calcium Content of MC3T3-E1 Pre-Osteoblasts

**Figure 3:**
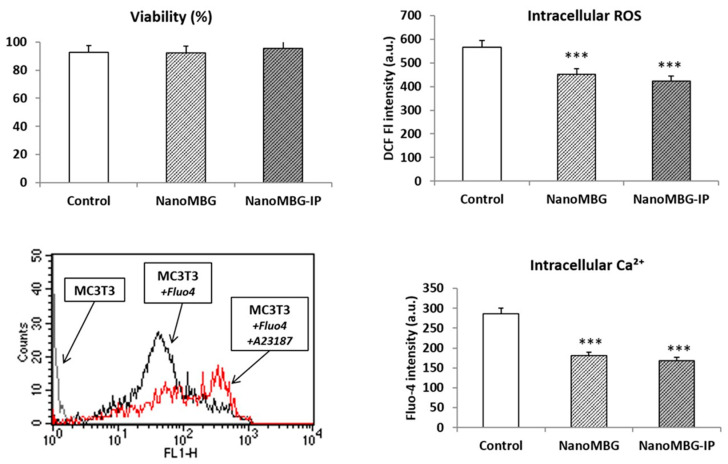
Effects of 50 μg/mL of nanoMBG and nanoMBG–IP nanospheres on viability, intracellular content of reactive oxygen species (ROS) and cytosolic calcium of MC3T3-E1 pre-osteoblasts, after 24 h of incubation. Control conditions without nanospheres were performed at the same time. Fluorescence profiles of control cells, cells with Fluo4 and cells with Fluo4 plus A23187 ionophore are shown in the lower-left figure. Statistical significance: *** *p* < 0.005.

Although no adverse effects on cell cycle were observed and IP evidenced a protective impact, the incorporation of nanoparticles could trigger an increment of the intracellular content of reactive oxygen species (ROS), oxidative stress, a decrease of cell viability and toxicity mechanisms [34]. On the other hand, bioactive mesoporous materials exhibit a high capability for releasing Ca^2+^ and other ions such as soluble silicate that can stimulate the proliferation and differentiation of osteoblasts [35,36,37], inducing bone regeneration due to the release of these two ions [38]. Considering all these facts, in the present work, we have evaluated the cell viability, intracellular content of ROS and cytosolic calcium of MC3T3-E1 pre-osteoblasts after treatment with nanoMBGs and nanoMBG–IPs. Control conditions without nanospheres were performed at the same time. Figure 3 shows the obtained results. The fluorescence profiles of control cells, cells with Fluo4 and cells with Fluo4 plus A23187 ionophore are also shown in the lower-left figure. The fluorescence increase observed after the addition of A23187 ionophore to the cells demonstrates the sensitivity of the assay. No viability changes but significant decreases of both intracellular ROS and calcium content were observed after incubation with 50 μg/mL of nanoMBGs and nanoMBG–IPs. These results evidence the absence of oxidative stress or toxicity caused by these nanospheres in MC3T3-E1 pre-osteoblasts after their uptake.

### 3.4. Effects of NanoMBGs and NanoMBG-IPs on Differentiation of MC3T3-E1 Pre-Osteoblasts

The set of results obtained and described in Section 3.2 and Section 3.3 evidence the excellent behavior in terms of cell viability and the absence of cytotoxicity of nanoMBGs and nanoMBG–IP. However, the application as osteoregenerative material requires the capability to stimulate the differentiation of the pre-osteoblasts toward the osteoblastic phenotype. The differentiation process of the MC3T3-E1 pre-osteoblasts includes three successive phases: (a) initial stage with active cell proliferation, but without expression of differentiation markers such as alkaline phosphatase (ALP) or mineral depositions; (b) intermediate stage with the maturation of the matrix and a high expression of ALP; and (c) final stage with matrix mineralization characterized by the presence of mineral depositions due to ALP activity [39,40]. On the other hand, ipriflavone is a synthetic drug that prevents osteoporosis by inhibiting bone resorption and maintaining bone thickness [24]. Thus, the use of nanoMBG–IPs for intracellular delivery of this drug could be a nanotherapeutic strategy to promote bone regeneration. In this context, we evaluate the impact of nanoMBG–IPs on MC3T3-E1 pre-osteoblast differentiation as a prototype of in vitro osteogenesis through the measurement of ALP activity and the quantification of matrix mineralization as key markers of MC3T3-E1 cell differentiation after 11 days of treatment with different doses of these nanospheres. Controls without nanospheres and with nanoMBGs, but without ipriflavone were performed at the same time.

**Figure 4:**
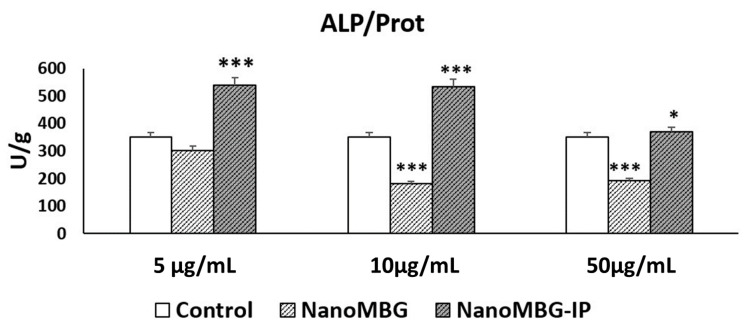
Effects of several doses of nanoMBGs and nanoMBG–IPs on MC3T3-E1 pre-osteoblast differentiation after 11 days, evaluated through the measurement of alkaline phosphatase (ALP) activity. Control conditions without nanospheres were performed at the same time. Statistical significance: *** *p* < 0.005, * *p* < 0.05.

Figure 4 shows that the cell incorporation of nanoMBG without ipriflavone induced a decrease of ALP activity compared to control cells after 11 days of incubation with 10 and 50 μg/mL. However, significant increases of ALP activity were observed after treatment with 5, 10 and 50 μg/mL of these nanospheres loaded with ipriflavone (nanoMBG–IPs), thus indicating the efficient intracellular release of IP and its positive in vitro effect on osteogenesis. The effect of the highest dose (50 μg/mL) of nanoMBG–IPs was lower than the obtained with 5 and 10 μg/mL of nanoMBG–IPs, evidencing the convenience of using lower doses than 50 μg/mL.

Increases of matrix mineralization were detected after the incubation with 50 μg/mL of nanoMBGs and nanoMBG–IPs for 11 days, but these effects were not statistically significant (Figure S4, Supplementary Materials), probably as a result of the lower precision and sensitivity of this test.

The ALP activity results demonstrate the efficient intracellular release of the drug from the nanoMBG–IPs and suggest their potential application as intracellular drug delivery systems in a nanotherapeutic strategy to promote bone regeneration.

Regarding the effects of other nanoparticles on MC3T3-E1 cell differentiation, in previous studies with this cell type and graphene oxide (GO) nanosheets, we observed that the treatment with 40 µg/mL of 400 nm PEG-GO for 3 days did not affect the differentiation process 12 days after the intracellular uptake of the nanomaterial [32].

### 3.5. Effects of NanoMBGs and NanoMBG-IPs on Interleukin 6 (IL-6) Production by MC3T3-E1 Pre-Osteoblasts

**Figure 5:**
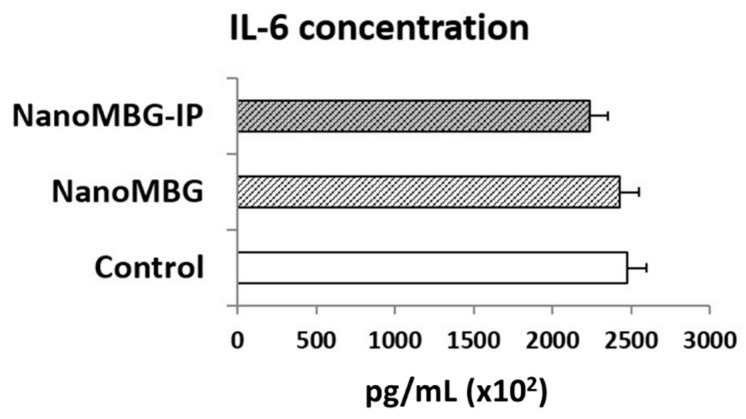
Effects of nanoMBGs and nanoMBG–IPs on interleukin 6 (IL-6) production by cultured MC3T3-E1 pre-osteoblasts after treatment with 50 μg/mL of nanospheres for 24 h. Control conditions without nanospheres were performed at the same time.

Despite having demonstrated the absence of cytotoxicity of these nanospheres and having observed their capability to promote pre-osteoblast differentiation, the inflammatory response that any kind of nanoparticles could elicit should be evaluated. In this sense, the detection *in vitro* of inflammatory cytokines provides valuable information about these potential clinical complications. IL-6 is produced by many cells, including osteoblasts, monocytes, macrophages and bone marrow mononuclear cells [41]. In bone, this cytokine induces osteoclast differentiation [42,43]. On the other hand, recent studies in a murine model have shown the IL-6 is related to the processes of revascularization and bone formation after ischemic osteonecrosis [44]. In the present work, we have quantified the levels of IL-6 secreted by cultured MC3T3-E1 pre-osteoblasts after incubation with nanoMBGs and nanoMBG–IPs. Figure 5 suggests that no significant changes of IL-6 secretion were detected after treatment with these nanospheres.

With respect to IL-6, it is important to note that this cytokine and tumor necrosis factor-alpha (TNF-α) play a key role in the inflammatory response, infection and stress [45]. In the present work, no significant changes of *in vitro* IL-6 secretion by pre-osteoblasts were detected after nanoMBG and nanoMBG–IP treatment, thus indicating that the local nanomaterial administration *in vivo* would not trigger the production of this pro-inflammatory cytokine and would not activate the innate immune system. These results agree with the switch of the M1 pro-inflammatory macrophage phenotype to the M2 reparative phenotype previously observed [29].

The results obtained so far evidence not only the excellent biocompatibility of nanoMBG–IP but also their capability to promote pre-osteoblasts differentiation towards osteoblast phenotype, thus confirming the osteogenic potential of nanoMBG–IP. In this sense, the intracellular release of the drug seems to play an important role in this process. The following experiments were carried out to shed some light on the mechanism that rules the incorporation of these nanoparticles within cells.

### 3.6. Uptake of NanoMBGs by MC3T3-E1 Pre-Osteoblasts

**Figure 6:**
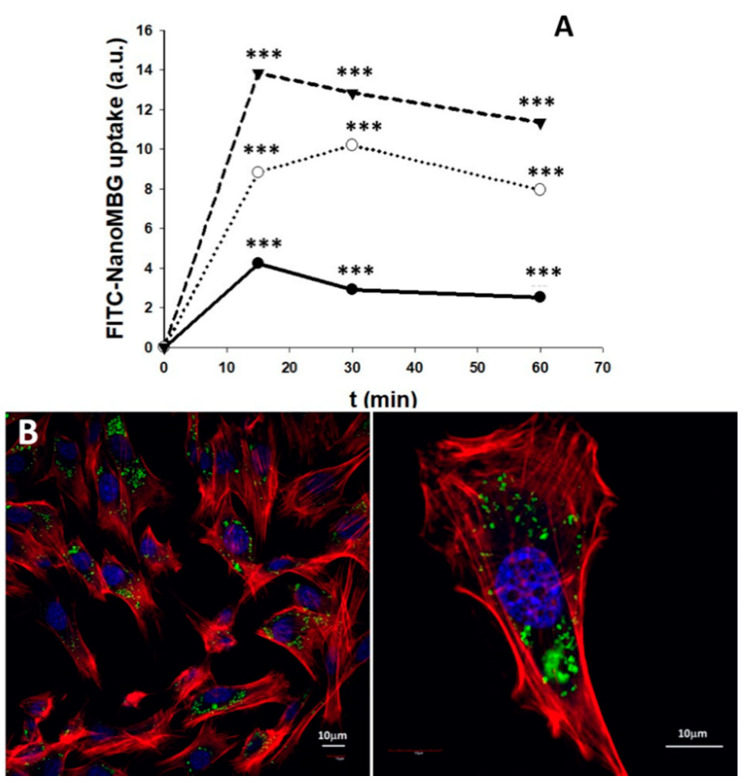
Intracellular uptake of nanoMBG nanospheres labeled with FITC by MC3T3-E1 pre-osteoblasts. (**A**) flow cytometric analysis of fluorescence intensity of cells with intracellular FITC–nanoMBG nanospheres after incubation with 10 (●), 30 (○) and 50 µg/mL (▼) for different times (15, 30 and 60 min). Statistical significance: *** *p* < 0.005. (**B**) Confocal microscopy images of MC3T3-E1 pre-osteoblasts after 24 h of incubation with 50 μg/mL of nanoMBG nanospheres labeled with fluorescein isothiocyanate (FITC). Nuclei were stained with DAPI (blue), F-actin filaments were stained with rhodamine-phalloidin (red), and FITC–nanoMBGs are observed in green.

In order to evaluate the nanoparticles uptake by pre-osteoblast cells, nanoMBG nanospheres were labeled with FITC. As a first approach, MC3T3-E1 pre-osteoblasts were cultured for 15, 30 and 60 min with 10, 30 and 50 µg/mL of FITC–nanoMBG. The cells were then detached, and the amount of cell-associated fluorescence was detected by flow cytometry as a measure of the intracellular uptake of these nanospheres. As can be observed in Figure 6A, the fluorescence intensity of osteoprogenitor cells after each treatment reveals a fast and dose-dependent FITC–nanoMBG uptake after 15 min. On the other hand, a decrease in fluorescence related to the intracellular content of these nanospheres was observed after 60 min of treatment with all the doses used (Figure 6A). This fact indicates that, after FITC–nanoMBG uptake, the exocytosis of this nanomaterial also occurs, according to the process described for other nanoparticles in mammalian cells [46].

For confocal microscopy studies, the dose of 50 µg/mL of FITC–nanoMBGs and 24 h time were chosen to observe if the intracellular uptake of this nanomaterial by MC3T3-E1 pre-osteoblasts could damage the cytoskeleton structure in these conditions of high dose and longer treatment time. Control cultures without this nanomaterial were performed at the same time. Figure 6B shows the abundance of nanospheres in the cytoplasm of the pre-osteoblasts and the integrity of their morphology. The results evidence that the incorporation of FITC–nanoMBGs did not induce changes in the pre-osteoblast cytoskeleton.

### 3.7. Endocytic Mechanisms for FITC–NanoMBG Entry into MC3T3-E1 Pre-Osteoblasts

**Figure 7:**
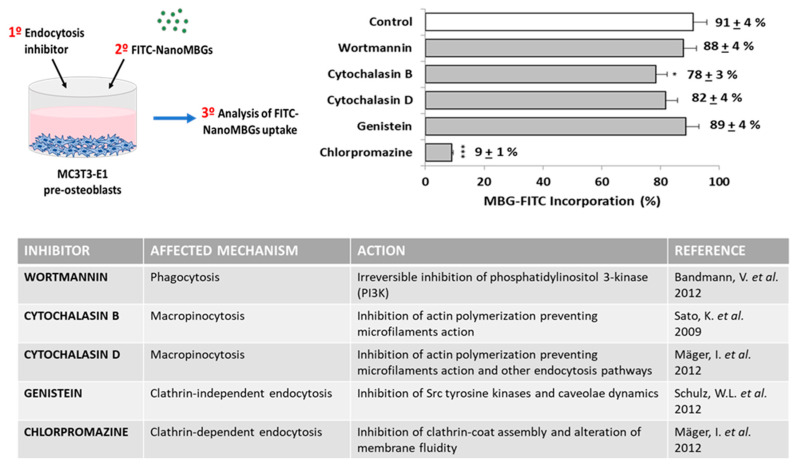
Inhibitory effects of several endocytosis inhibitors on FITC–nanoMBG uptake by MC3T3-E1 pre-osteoblasts. Cells were incubated with each inhibitor for 2 h, the medium was then removed, and the cultures were treated with 50 µg/mL FITC–nanoMBGs for 2 h. Statistical significance: * *p* < 0.05, *** *p* < 0.005.

Five specific endocytosis inhibitors were added into the culture wells before the nanomaterial addition in order to identify the endocytic mechanisms by which these FITC–nanoMBG nanospheres are incorporated within the MC3T3-E1 cells. This indirect method consists of pretreating the cells with different inhibitors that specifically block a certain mechanism of endocytosis. In this way, when the inhibitor used reduces the entry of the nanospheres, we can know that this mechanism that has been blocked constitutes an entry route. On the contrary, if the inhibitor does not decrease the entry of the nanospheres, we will know that the mechanism that is blocking the inhibitor is not involved in the entry of the nanospheres. Figure 7 shows a scheme of the assay, a table with the mechanism affected by each inhibitor (its specific action and the corresponding reference) and a graph with the effects of these agents on the FITC–nanoMBG uptake. Previous studies allowed us to choose the dose of the different inhibitors [47,48,49,50,51]. The results showed two incorporation mechanisms for FITC–nanoMBG entry into MC3T3-E1 pre-osteoblasts.

Cytochalasins B and D, which block actin polymerization and inhibit macropinocytosis, reduce the FITC–nanoMBG incorporation by pre-osteoblasts, although only the effect of Cytochalasin B was significant (*p* < 0.05, Figure 3). Chlorpromazine is an inhibitor of clathrin-dependent mechanisms, and this agent produced a very pronounced diminution (*p* < 0.005) of FITC–nanoMBG incorporation by MC3T3-E1 cells, thus indicating that the clathrin-dependent endocytic mechanism is the main route implicated in the entry of these nanospheres into pre-osteoblasts. Previous studies with nanosheets of graphene oxide and Saos-2 osteoblasts evidenced that these nanosheets can enter in mature osteoblasts through pathways dependent on microtubules [51]. It is important to note that the mechanisms of entry of nanomaterials into cells depend on the cell type and the characteristics of the nanoparticles. In the present study, the treatment with either wortmannin or genistein did not trigger significant changes on FITC–nanoMBG incorporation by pre-osteoblasts. Wortmannin blocks the activity of phosphoinositide 3-kinase (PI3K) and phosphoinositide 4-kinase (PI4K) [47], with key roles in cell development and growth as adhesion, apoptosis, cytoskeletal organization, motility, proliferation, thus preventing phagocytosis mechanisms [52]. Genistein blocks Src tyrosine kinases and the dynamics of caveolae [50], and no differences were observed in the uptake of these nanospheres when it was present in the cell culture. Since wortmannin and genistein did not reduce FITC–nanoMBG uptake by pre-osteoblasts, we can conclude that neither phagocytosis nor caveolae-mediated incorporation is routes implicated in the *in vitro* uptake of these nanospheres by MC3T3-E1 cells.

## 4. Conclusions

The novelty of this work is the knowledge of the effects of ipriflavone-loaded mesoporous nanospheres on the differentiation of bone-forming cells. In previous studies, the effects of these nanoparticles on already differentiated osteoblasts in coculture with osteoclasts were analyzed [25], but until now, their effects on osteoprogenitor cells were unknown. Another of the novel objectives of the present work was to understand the mechanisms by which these nanoparticles are incorporated into osteoprogenitor cells. The obtained results demonstrate active incorporation of nanoMBG–IPs by MC3T3-E1 pre-osteoblasts that stimulates their differentiation into mature osteoblast phenotype with increased alkaline phosphatase activity, thus indicating the efficient intracellular release of the drug and its positive *in vitro* effect on osteogenesis. The main mechanism by which FITC-Nano-MBGs enter pre-osteoblasts is the clathrin-dependent route, although these nanospheres can also enter through micropinocytosis. The present work reveals the absence of cytotoxicity of nanoMBG–IPs and their great potential as a nanotherapeutic strategy for the intracellular delivery of ipriflavone to promote osteogenesis in the periodontal defects. On the other hand, having demonstrated the intracellular incorporation of these nanospheres and their effective intracellular release of ipriflavone, this study represents the starting point for the use of these nanospheres as carriers of very diverse drugs (antibiotics, anti-inflammatory, antiresorptive and osteogenic drugs) not only for periodontal defects but also for infections and inflammatory processes such as those that occur in periodontitis.

## Figures and Tables

**Table 1:** Textural properties for nanoMBG and nanoMBG–IP spheres measured by N_2_ adsorption.

Sample	Surface Area (m^2^·g^−1^)	Pore Volume (cm^3^·g^−1^)	Pore Size (nm)
**nanoMBG**	543.6	0.435	−2.5 nm
**nanoMBG–IP**	14.4	0.057	NA

## Supplementary Materials

The following are available online at https://www.mdpi.com/2079-4991/10/12/2573/s1, Figure S1: FTIR spectrum of ipriflavone; Figure S2: Thermogravimetric analysis before (nanoMBG) and after loading with ipriflavone (nanoMBG–IP); Figure S3: Effects of nanoMBGs and nanoMBG–IPs (50 μg/mL) on cell size and complexity of MC3T3-E1 pre-osteoblasts after 24 h of treatment with 50 μg/mL of nanospheres. Control conditions without nanospheres were performed at the same time; Figure S4: Effects of nanoMBGs and nanoMBG–IPs on matrix mineralization by MC3T3-E1 pre-osteoblasts after 11 days of treatment with 50 μg/mL of nanospheres by Alizarin Red staining. Control conditions without nanospheres were performed at the same time.
