# Supplementary material for: Ipriflavone-Loaded Mesoporous Nanospheres with Potential Applications for Periodontal Treatment
Source: Nanomaterials (Basel). 2020 Dec 21;10(12):2573. doi: 10.3390/nano10122573 (PMC7767486; doi:10.3390/nano10122573)
Supplement: Supplementary file 1 [file nanomaterials-10-02573-s001.pdf]

Supplementary Materials

# Ipriflavone-Loaded Mesoporous Nanospheres with Potential Applications for Periodontal Treatment

**Laura Casarrubios [1], Natividad Gómez-Cerezo [2,3], María José Feito [1], María Vallet-Regí [2,3,*], Daniel Arcos [2,3,*] and María Teresa Portolés [1,3,*]**

1 Departamento de Bioquímica y Biología Molecular, Facultad de Ciencias Químicas, Universidad Complutense de Madrid, Instituto de Investigación Sanitaria del Hospital Clínico San Carlos (IdISSC), 28040 Madrid, Spain; laura.casarrubios.molina@gmail.com (L.C.); mjfeito@ucm.es (M.J.F.)

2 Departamento de Química en Ciencias Farmacéuticas, Facultad de Farmacia, Universidad Complutense de Madrid, Instituto de Investigación Sanitaria Hospital 12 de Octubre i+12, Plaza Ramón y Cajal s/n, 28040 Madrid, Spain; magome21@ucm.es

3 CIBER de Bioingeniería, Biomateriales y Nanomedicina, CIBER-BBN, 28040 Madrid, Spain

* Correspondence: vallet@ucm.es (M.V.-R.); arcosd@ucm.es (D.A.); portoles@quim.ucm.es (M.T.P.)

**S.1 Synthesis of mesoporous $SiO_2$–CaO nanospheres**

The synthesis of mesoporous $SiO_2$–CaO nanospheres (nanoMBGs) was carried as follows: 80 mg of PS-b-PAA were dissolved in 16 mL of tetrahydrofuran (THF) at room temperature. This solution was subsequently poured in a CTAB solution, previously prepared by dissolving 160 mg of CTAB in 74 mL of deionized water and 2.4 mL of ammonia (28% w/v) and gently stirred in an incubator at 37°C. The mixture was magnetically stirred for 20 min, and a solution of 25 mL of TEP in 1.6 mL of ethanol was added drop-by-drop and stirred for another 20 min. Thereafter, a solution of 125 mg of $Ca(NO_3)·4H_2O$ in 1.6 mL of water was also added and stirred for 10 min, and, finally, the silica source was incorporated as a solution of 0.52 mL of TEOS in 1.6 mL of ethanol. After stirring for 24 h, the product was collected by centrifugation at 10,000 rpm for 10 min and washed three times with a mixture of ethanol–water (50:50). The product was dried at 30 °C under vacuum conditions, and the organic template was removed by calcination at 550 °C for 4 h with a heating rate of 1 °C $min^{-1}$. All reactants were purchased from Sigma-Aldrich (St. Louis, MO, USA).

**S.2 Fluorescein isothiocyanate (FITC) labeling**

For fluorescein isothiocyanate (FITC) labeling of nanoMBGs, 50 mg of nanospheres were degasified at 80 °C for 24 h and resuspended in 4 mL of toluene. Moreover, 44.3 L of aminopropyl triethoxysilane (APTES) was dissolved in 0.5 mL of ethanol and reacted with 0.6 mg of fluorescein isothiocyanate for 5 h. This solution was added dropwise on the nanoMBG suspension and reacted at 80 °C for 12 h under a nitrogen atmosphere. Finally, fluorescein-labeled nanoMBGs (FITC–nanoMBGs) were thoroughly washed and centrifuged several times to remove the excess of fluorescein non-covalently adsorbed to the nanospheres.

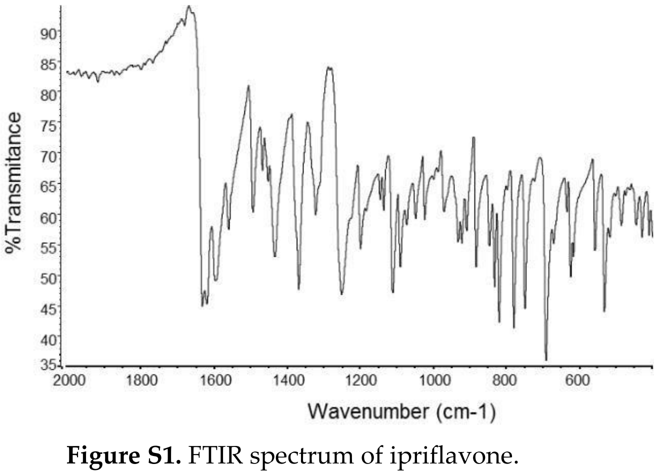


**Figure S1.** FTIR spectrum of ipriflavone.

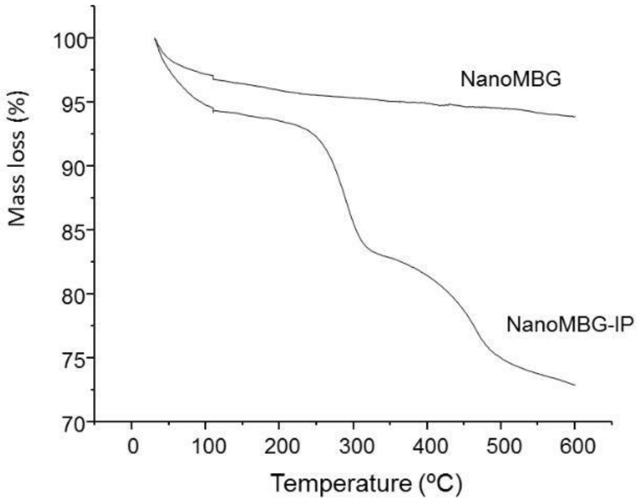


**Figure S2.** Thermogravimetric analysis before (nanoMBG) and after loading with ipriflavone (nanoMBG–IP).

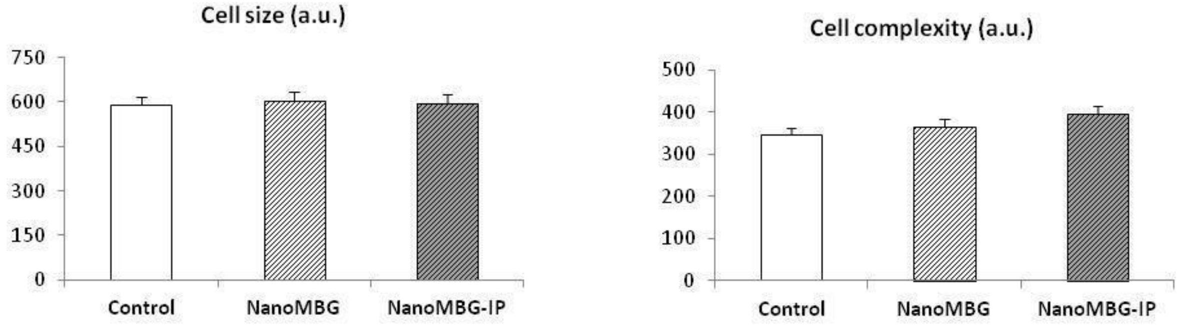


**Figure S3.** Effects of nanoMBGs and nanoMBG–IPs (50 μg/mL) on cell size and complexity of MC3T3-E1 pre-osteoblasts after 24 h of treatment with 50 μg/mL of nanospheres. Control conditions without nanospheres were performed at the same time.

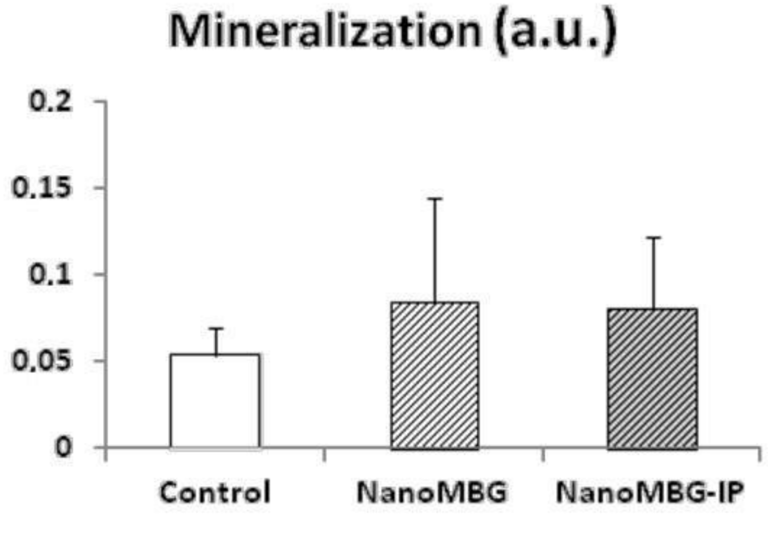


**Figure S4.** Effects of nanoMBGs and nanoMBG–IPs on matrix mineralization by MC3T3-E1 pre-osteoblasts after 11 days of treatment with 50 μg/mL of nanospheres by Alizarin Red staining. Control conditions without nanospheres were performed at the same time.
